# Genetically predicted immune cells mediate the association between gut microbiota and autoimmune liver diseases

**DOI:** 10.3389/fmicb.2024.1442506

**Published:** 2024-12-16

**Authors:** Jikang Zhang, Yiqi Hu, Jin Xu, Hua Shao, Qingping Zhu, Hao Si

**Affiliations:** ^1^General Surgery Department, The Second Affiliated Hospital of Nanjing University of Chinese Medicine, Nanjing, China; ^2^Digestive Endoscopic Treatment Center, The Second Affiliated Hospital of Nanjing University of Chinese Medicine, Nanjing, China; ^3^General Surgery Department, Nanjing Pukou District Traditional Chinese Medicine Hospital, Nanjing, China

**Keywords:** Mendelian randomization, autoimmune hepatitis, primary biliary cholangitis, primary sclerosing cholangitis, immune cells, gut microbiota

## Abstract

**Background:**

Increasing evidence suggests an association between gut microbiota and Autoimmune Liver Diseases (AILDs). However, causal inference remains controversial due to confounding bias in observational studies. Additionally, there is currently no clear evidence indicating that immune cells act as intermediate phenotypes in the pathogenesis of AILDs. This study utilizes the Mendelian Randomization (MR) method to investigate the causal relationships among gut microbiota, immune cells, and AILDs.

**Methods:**

Initially, we conducted a two-sample MR analysis to predict the causal relationships among 412 gut microbiota, 731 immune phenotypes, and AILDs. Subsequently, a series of sensitivity analyses were performed to validate the initial MR results and reverse MR analysis was conducted to exclude reverse causality. Finally, a two-step MR analysis was utilized to quantify the proportion of the impact of gut microbiota on AILDs mediated by immune cells.

**Results:**

Following rigorous MR analysis, our findings indicate that increased involvement of the gut microbiome in the *superpathway of L-tryptophan biosynthesis* is positively associated with an elevated risk of Autoimmune Hepatitis (AIH). The effect is partially mediated by the *CD14+ CD16+ monocyte Absolute Count*, which accounts for 17.47% of the total effect. Moreover, the *species Ruminococcus obeum* appears to mediate the development of Primary Sclerosing Cholangitis (PSC) through *CD62L-CD86+ myeloid Dendritic Cell %Dendritic Cell*, contributing to 32.47% of the total observed effect.

**Conclusion:**

Our study highlights the potential mediating mechanisms of immune cells in the causal relationship between the gut microbiome and AILDs. These insights provide a foundation for developing preventive strategies for AILDs in clinical practice.

## 1 Introduction

Autoimmune Liver Diseases (AILDs) represent a spectrum of disorders characterized by the immune system’s persistent and progressive assault on hepatic cells. This category encompasses Autoimmune Hepatitis (AIH), Primary Biliary Cholangitis (PBC), and Primary Sclerosing Cholangitis (PSC) (Fischer and Goltz, 2020). Individuals afflicted with AILDs commonly experience symptoms like fatigue, jaundice, and abdominal discomfort, which may progress to severe complications such as cirrhosis and liver failure if not adequately managed (Muratori et al., 2023). The incidence of AILDs has historically been low worldwide, but it has been gradually increasing in recent years, presenting substantial challenges to both affected individuals and healthcare infrastructure. Moreover, these diseases are frequently associated with extraliver conditions, including arthralgia and thyroiditis, affecting a considerable proportion of patients (Mihai et al., 2024). This widespread impact underscores the urgent need for refined diagnostic approaches and effective treatment strategies in the management of AILDs (Kardashian et al., 2023).

Increasing research emphasizes the critical role of the gut microbiome in the development of AILDs via the gut-liver axis (Qian et al., 2023). This involves mechanisms such as gut barrier dysfunction, immune regulation, metabolic products, and inflammatory factors. With advances in immunology, it has also been recognized that the imbalance of gut microbiota and its metabolic products are key signals inducing immune responses in the liver tissue of AILDs (Schneider et al., 2023; Zheng et al., 2021). Studies suggested that Pien Tze Huang (PTH) stimulates beneficial bacteria, increasing Regulatory T cell (Treg)/myeloid Regulatory T cell (mTreg) cells and Interleukin (IL)-10 production, inhibiting Toll-like receptor (TLR)4/Nuclear Factor-κB (NF-κB) and C-X-C motif chemokine ligand 16 (CXCL16)/C-X-C motif chemokine receptor 6 (CXCR6) signaling pathways, and reducing liver pathology in AIH mice (Oldereid et al., 2024). In PBC mouse models, early gut microbiome changes induce Bile duct Epithelial Cell (BEC) apoptosis via TLR2, recruiting CD8 T cells and causing a vicious cycle (Wang et al., 2022). Quraishi et al. discovered reduced gut microbiota diversity in PSC patients, potentially leading to abnormal homing of gut-specific lymphocytes and gut permeability, causing mucosal immune dysregulation (Quraishi et al., 2017). However, current research primarily focuses on animal experiments and small-scale clinical studies aimed at elucidating the potential correlation between gut microbiota and AILDs. These studies rarely form a coherent pathological mechanism, and direct evidence of the critical role of immune cells in the pathogenesis of AILDs is still lacking.

Mendelian Randomization (MR) is a method used in epidemiology and genetics to assess the causal impact of exposures on outcomes, offering significant advantages in determining causality and directionality (Larsson and Burgess, 2021). We obtained the latest summary statistics for 412 gut microbiota and 731 immune cells from large public genome-wide association study (GWAS) consortia to perform two-sample and mediation MR analyses. This approach evaluates the causal relationships between the gut microbiome and AILDs and explores the potential mediating role of immune cells. These insights provide valuable understanding for future genetic and therapeutic research related to the gut microbiome and AILDs.

## 2 Materials and methods

### 2.1 Study design

**Figure 1:**
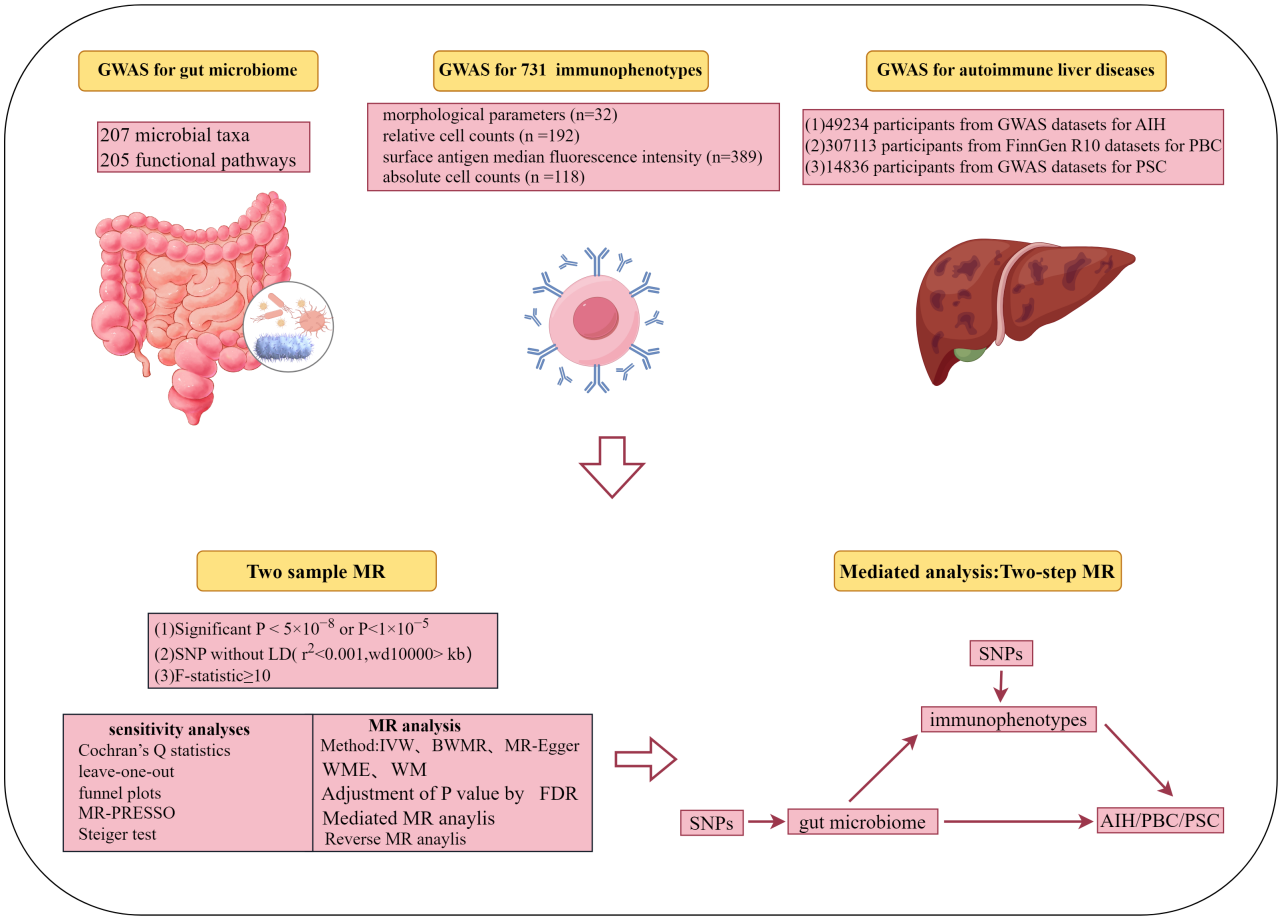
Flow chart of the study.

We conducted a two-sample and mediation MR study to explore the causal relationships between gut microbiota, immune cells, and AILDs, as displayed in Figure 1. When conducting MR analyses, instrumental variables (IVs) must satisfy three core principles: (i) genetic variants used as IVs must be significantly associated with gut microbiota or immune cells; (ii) genetic variants must not be influenced by other confounding factors; and (iii) genetic variants must affect AILDs only through gut microbiota or immune cells.

### 2.2 Data sources

The summary statistics for gut microbiota were obtained from the Dutch microbial GWAS, which included metagenomic sequencing data from 7,738 individuals in the northern Netherlands L (Lopera-Maya et al., 2022). This GWAS explored the relationship between host genetics and the gut microbiome by analyzing 207 microbial taxa and 205 functional pathways.

The genetic dataset analyzing 731 distinct immunophenotypes from 3,757 Sardinians was sourced from a GWAS (accession numbers GCST0001391 to GCST0002121) (Orrù et al., 2020). This dataset includes 539 immune traits and 192 relative count levels, encompassing morphological parameters, median fluorescence intensities of surface antigens, and cell counts. These immunophenotypes elucidate the complex genetic regulation of immune cells at the cell-subtype level in autoimmune diseases.

The dataset for AIH was sourced from a GWAS (GCST90018785) and includes 821 cases and 484,413 controls, all of European ancestry (Sakaue et al., 2021). We acquired genetic data for PBC from the FinnGen R10 database, comprising 609 cases of PBC out of a total of 306,504 participants. The summary statistics data are accessible through the following Google Cloud Storage link: https://r10.finngen.fi/. This dataset provides a comprehensive genetic insight into PBC, aiming to identify associated genetic variants. The PSC dataset, obtained by Sun-Gou Ji and colleagues from the International PSC Study Group, represents the largest PSC GWAS dataset to date (Ji et al., 2017). In addition, it focuses on the genetic determinants associated with PSC, comprising 2,871 cases and 12,019 controls. Moreover, the analysis has been adjusted for potential confounders such as gender, age, and genotyping batches. All original data in this study were sourced from publicly available databases and previous studies, all of which had received ethical approval.

### 2.3 Instrumental variables selection

To obtain a sufficient number of Single Nucleotide Polymorphisms (SNPs) related to the gut microbiota, we set the selection criteria for IVs at *p* < 1 × 10^−5^. This threshold is consistent with similar criteria utilized in published studies (Qu et al., 2024). Given the sufficient number of SNPs for immune cell phenotypes, we applied a more stringent threshold of *p* < 5 × 10^−8^ to select SNPs closely associated with them. Additional screening criteria included the following: First, screening for independent SNPs was conducted through Linkage Disequilibrium (LD) analyses, utilizing a threshold of r^2^ < 0.001 and a physical distance of 10,000 kb (Palmer et al., 2012; Levin et al., 2020). Second, to reduce weak instrument bias and ensure a strong correlation with exposure, SNPs with an F-statistic greater than 10 were selected as IVs (Gill et al., 2019).

### 2.4 Statistical analysis strategy

In this study, we employed five distinct methods to assess the bidirectional causal relationships between the gut microbiome, immune cells, and AILDs. These methods included Inversevariance Weighted (IVW) (Burgess et al., 2013), Bayesian Weighted Mendelian Randomization (BWMR), MR-Egger, Weighted Median (WME), and Weighted Mode (WM) (Burgess et al., 2015; Bowden et al., 2015). The IVW method served as our primary approach, offering high efficiency and accuracy, simplicity, the ability to reduce the impact of individual SNP bias, and broad applicability. It is widely used in epidemiological research. Note that results were considered statistically significant when the IVW *p*-value was less than 0.05. At the same time, BWMR offered the advantage of addressing pleiotropy and weak instrument biases through Bayesian weighting while enhancing analysis efficiency with an advanced Variational Expectation Maximization algorithm and was, therefore, used as a supplementary method to IVW (Bowden et al., 2016). Additionally, to control for potential false positives in large-scale omics studies, we applied the False Discovery Rate (FDR) to manage statistical bias resulting from multiple comparisons while maintaining statistical power (Ma et al., 2024). Notably, associations were considered significant when FDR < 0.05.

To address potential issues such as pleiotropy and heterogeneity in our two-sample MR analyses, we conducted a series of sensitivity analyses. Heterogeneity, which may arise due to variations in experimental conditions, selected populations, and SNPs, was assessed using Cochran’s Q statistic, with a *p*-value >0.05 indicating no significant heterogeneity (Zhao et al., 2020). The MR-Egger intercept method was employed to evaluate pleiotropy, with a *p*-value >0.05 suggesting the absence of pleiotropy (Burgess et al., 2015). Additionally, the MR-PRESSO method was used to filter out SNPs that might introduce bias, thereby enhancing the reliability of causal inferences (Verbanck et al., 2018). Finally, leave-one-out analysis and funnel plots were utilized to ensure the consistency of the results. To further enhance the robustness of the IVs extracted, we employed the Steiger test method to mitigate reverse causation (Hemani et al., 2017). It is worth mentioning that, when the number of available SNPs was less than three, limiting the scope of comprehensive MR analysis, these results were excluded from the analysis to ensure robustness and reliability of the findings. This approach was taken to maintain the integrity of the MR analysis and to focus on results derived from a sufficient number of SNPs for meaningful causal inference. Additionally, to further mitigate the interference of reverse causality and ensure the reliability of our research findings, we used AILDs as the exposure. Significantly associated SNPs (*p* < 5 × 10^−8^) with AILDs were selected as IVs. Thus, we conducted reverse MR studies using gut microbiota or immune cells as the outcomes.

Exploration of the potential mediating mechanisms of immune cells was conducted through a two-step MR mediation analysis. Initially, univariable MR analysis was employed to estimate the effect of exposure on the mediator, obtaining β1. Subsequently, multivariable MR analysis was used to estimate the effect of each mediator on the outcome, obtaining β2. Meanwhile, the indirect effect was estimated by multiplying these two regression estimates (β1 × β2) (Carter et al., 2021). This methodology enabled the determination of the proportion of mediation by immune cells in the causal relationship between gut microbiota and AILDs. All analyses were conducted using “TwoSampleMR,” “MR-PRESSO,” and “frostplot,” a package in the R software (R version 4.3.2).

## 3 Results

### 3.1 Instrument variables included in analysis

Detailed information on the final SNPs meeting the requirements of our study for each bacterial trait or functional pathway is provided in Supplementary Table S1. All SNPs used in our analysis had *F*-values exceeding 10.

### 3.2 MR and sensitivity analysis of gut microbiota and AILDs

#### 3.2.1 AIH

**Figure 2:**
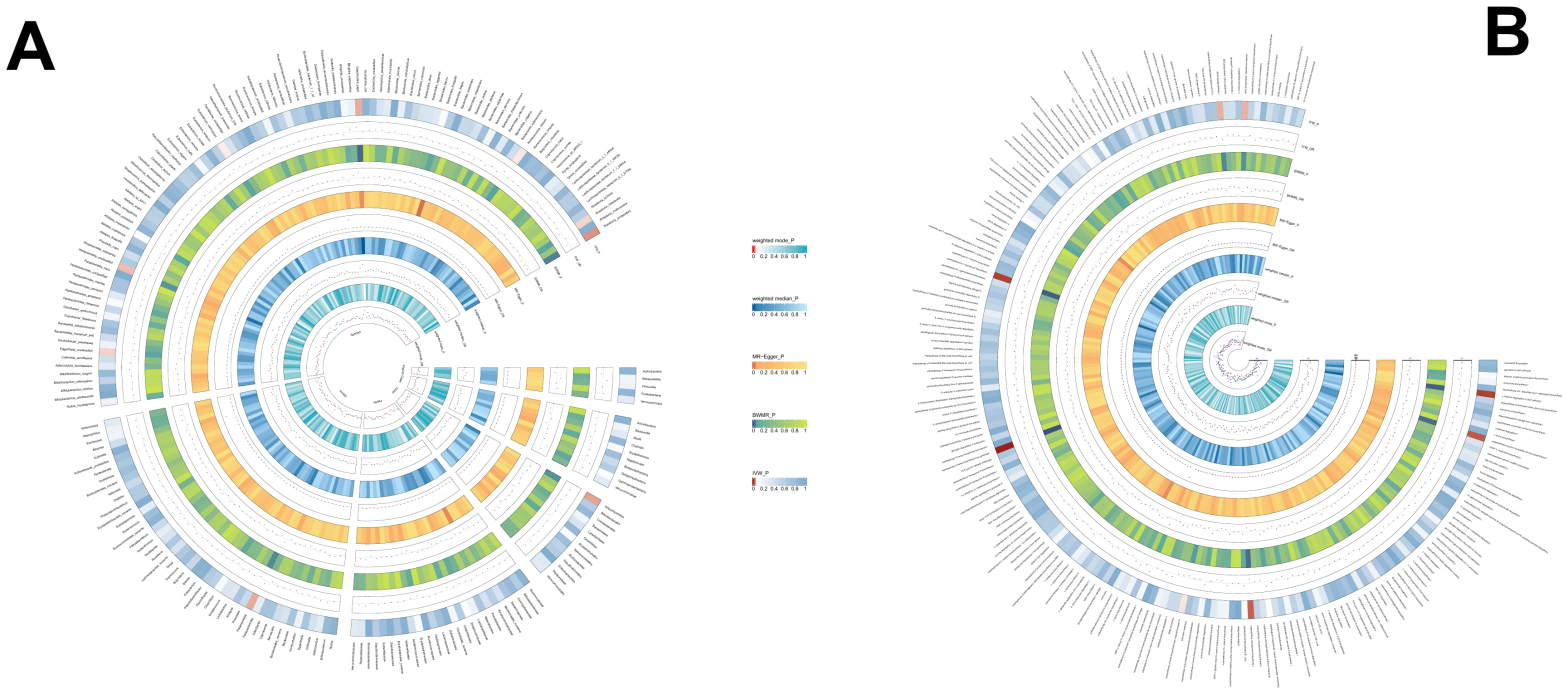
**(A)** The causal effect of the gut microbiota abundance on AIH. **(B)** The causal effect of the gut bacterial pathway abundance on AIH.

Through rigorous MR analysis, using the IVW method as our primary analytical strategy, we identified 16 gut microbial features associated with AIH, including 8 microbial taxa and 8 functional pathways (Figure 2; Supplementary Table S2). However, after FDR correction, no gut microbiota were significantly associated. *Superpathway of heme biosynthesis from glutamate* (OR = 0.552, *p* < 0.001), *superpathway of polyamine biosynthesis I* (OR = 0.557, *p* = 0.014), *Species.Roseburia unclassified* (OR = 0.788, *p* = 0.025) were the top three features negatively associated with AIH. Conversely, *Order.Actinomycetales* (OR = 1.533, *p* = 0.029), *superpathway of L-tryptophan biosynthesis* (OR = 1.380, *p* = 0.006), *L-arginine degradation II* (*AST pathway*) (OR = 1.490, *p* = 0.006) were positively associated with AIH.

#### 3.2.2 PBC

**Figure 3:**
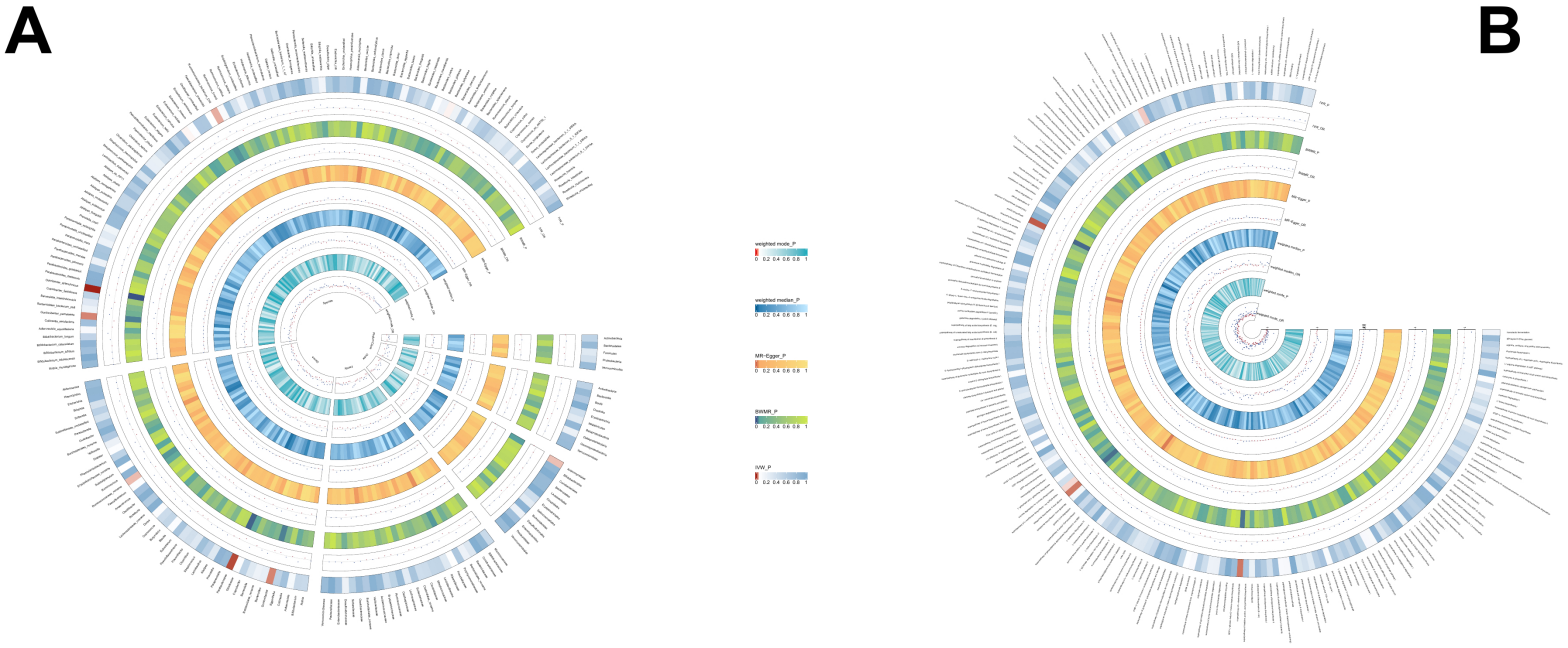
**(A)** The causal effect of the gut microbiota abundance on PBC. **(B)** The causal effect of the gut bacterial pathway abundance on PBC.

We identified 14 gut microbial features associated with PBC, including 9 microbial taxa and 5 functional pathways (detailed results are displayed in Figure 3; Supplementary Table S3). However, after FDR correction, no gut microbiota were significantly associated. *Genus.Parabacteroides* (OR = 0.562, *p* = 0.009), *Genus.Gordonibacter* (OR = 0.739, *p* = 0.022), *Species.Gordonibacter pamelaeae*: (OR = 0.740, *p* = 0.022) were the top three features negatively associated with PBC. Conversely, *Species.Odoribacter splanchnicus* (OR = 1.779, *p* = 0.001), *preQ₀ biosynthesis* (OR = 1.679, *p* = 0.013), and other features were positively associated with PBC.

#### 3.2.3 PSC

**Figure 4:**
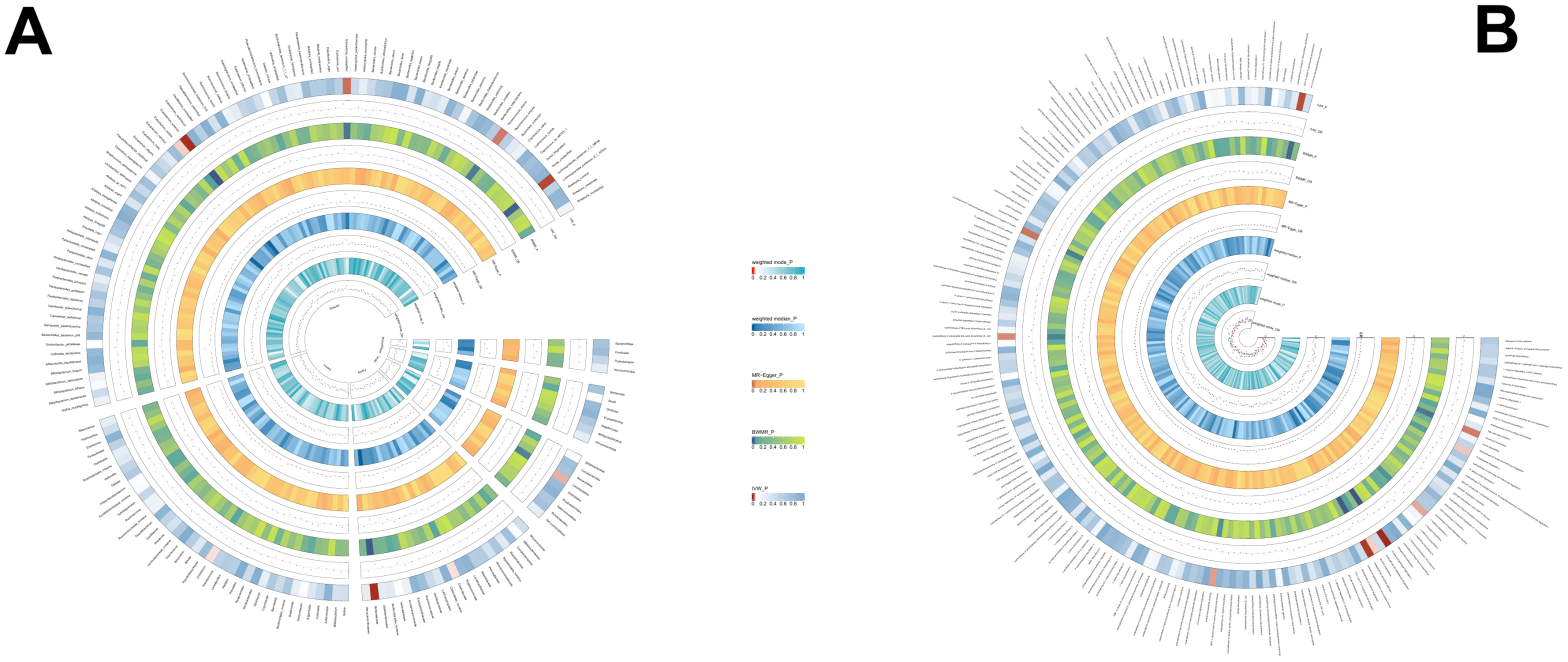
**(A)** The causal effect of the gut microbiota abundance on PSC. **(B)** The causal effect of the gut bacterial pathway abundance on PSC.

Using the same method, we identified 19 gut microbial features associated with PSC, including 9 microbial taxa and 10 functional pathways (detailed results are illustrated in Figure 4; Supplementary Table S4). However, after FDR correction, no gut microbiota was significantly associated. *Lactose and galactose degradation I*: (OR = 0.651, *p* = 0.002), *Family.Sutterellaceae*: (OR = 0.505, *p* = 0.003)*, Species.Eubacterium siraeum*: (OR = 0.545, *p* = 0.003) were the top three features negatively associated with PSC. Conversely, *Species.Lachnospiraceae bacterium_7_1_58FAA*: (OR = 1.490, *p* = 0.008), *Species.Escherichia unclassified* (OR = 1.500, *p* = 0.017), *mixed acid fermentation pathway* (OR = 1.864, *p* = 0.020), and other features were positively associated with PSC.

### 3.3 MR and sensitivity analysis of immune cells and AILDs

#### 3.3.1 AIH

**Figure 5:**
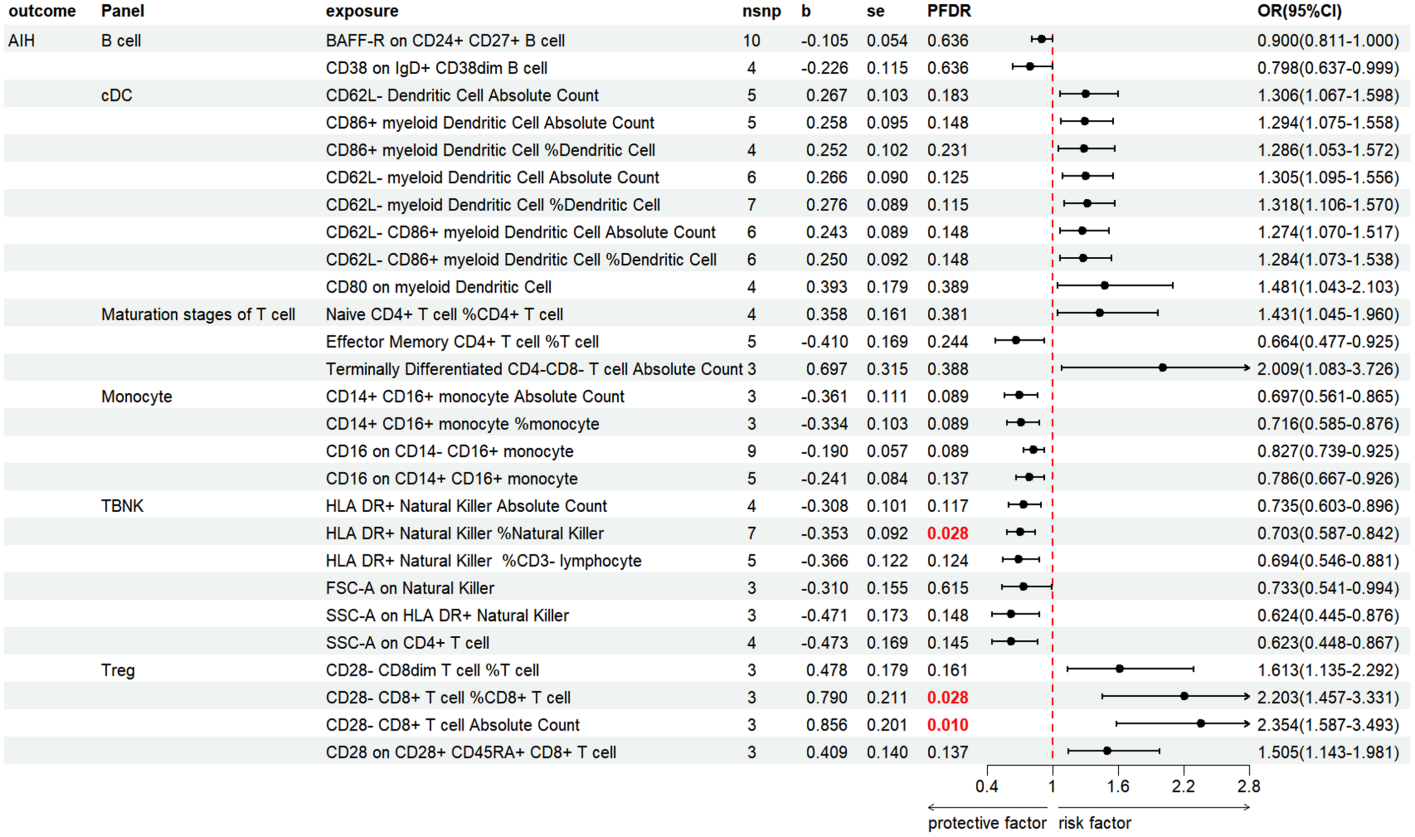
Forest plot illustrates the positive IVW-MR results of causal links between immune cells and AIH.

We discovered that 27 immune cell phenotypes significantly affected AIH, as evidenced by an IVW *p*-value of less than 0.05 (including 8cDC, 6TBNK, 4Monocyte, 4Treg, 3Maturation stages of T cell, 2B cell) (Figure 5; Supplementary Table S5). By FDR correction (PFDR <0.05), we discovered one immunophenotype to be protective against AIH: *HLA DR+ Natural Killer %Natural Killer* (OR = 0.703, 95%CI 0.587 ~ 0.842, *p* = 0.000134, PFDR = 0.028). In addition, we discovered that there are two immunophenotypes that have dangerous effects on AIH:*CD28-CD8+ T cell %CD8+ T cell* (OR = 2.203, 95%CI 1.457 ~ 3.331, *p* = 0.00018, PFDR = 0.028), *CD28-CD8+ T cell Absolute Count* (OR = 2.354, 95%CI 1.587 ~ 3.493, *p* = 2.107 × 10^−5^, PFDR = 0.0097).

#### 3.3.2 PBC

**Figure 6:**
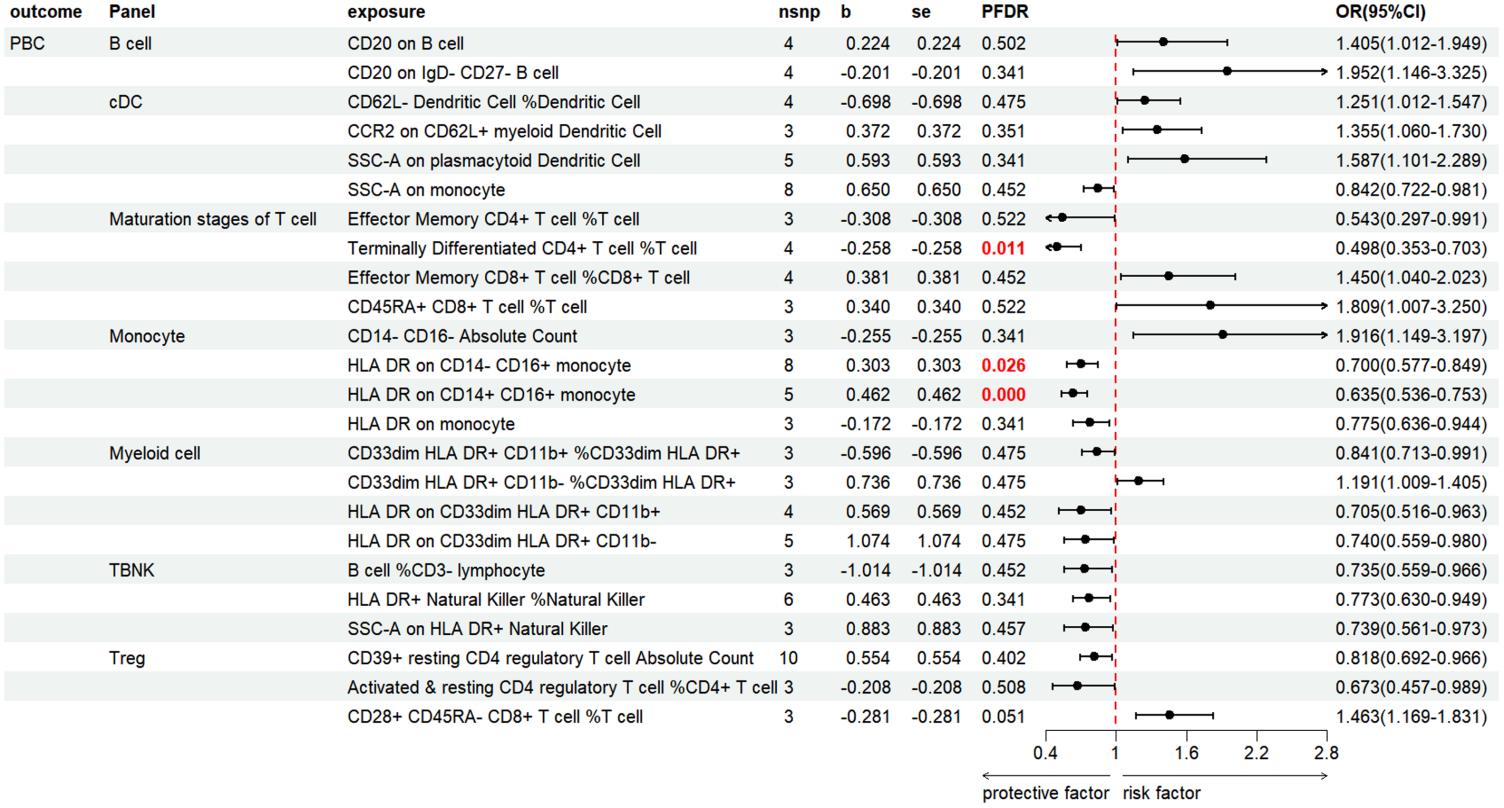
Forest plot shows the positive IVW-MR results of causal links between immune cell and PBC.

Based on the IVW method, the results indicate that 24 immune cell phenotypes significantly influence PBC. This includes 2B cell, 4cDC, 4Maturation stages of T cell, 4Monocyte, 4Myeloid cell, 3TBNK, 3Treg (Figure 6; Supplementary Table S6). By FDR correction (PFDR <0.05), we discovered three immunophenotypes to be protective against PBC: *Terminally Differentiated CD4+ T cell %T cell* (OR = 0.498, 95% CI 0.353 ~ 0.703, *p* = 7.331 × 10^−5^, PFDR = 0.0108). *HLA DR on CD14-CD16+ monocyte* (OR = 0.700, 95% CI 0.577 ~ 0.849, *p* = 0.00029, PFDR = 0.026). *HLA DR on CD14+ CD16+ monocyte* (OR = 0.635, 95% CI 0.536 ~ 0.753, *p* = 1.700 × 10^−7^, PFDR = 7.513 × 10^−5^). However, no immnophenotypes were found to be positively associated with PBC.

#### 3.3.3 PSC

**Figure 7:**
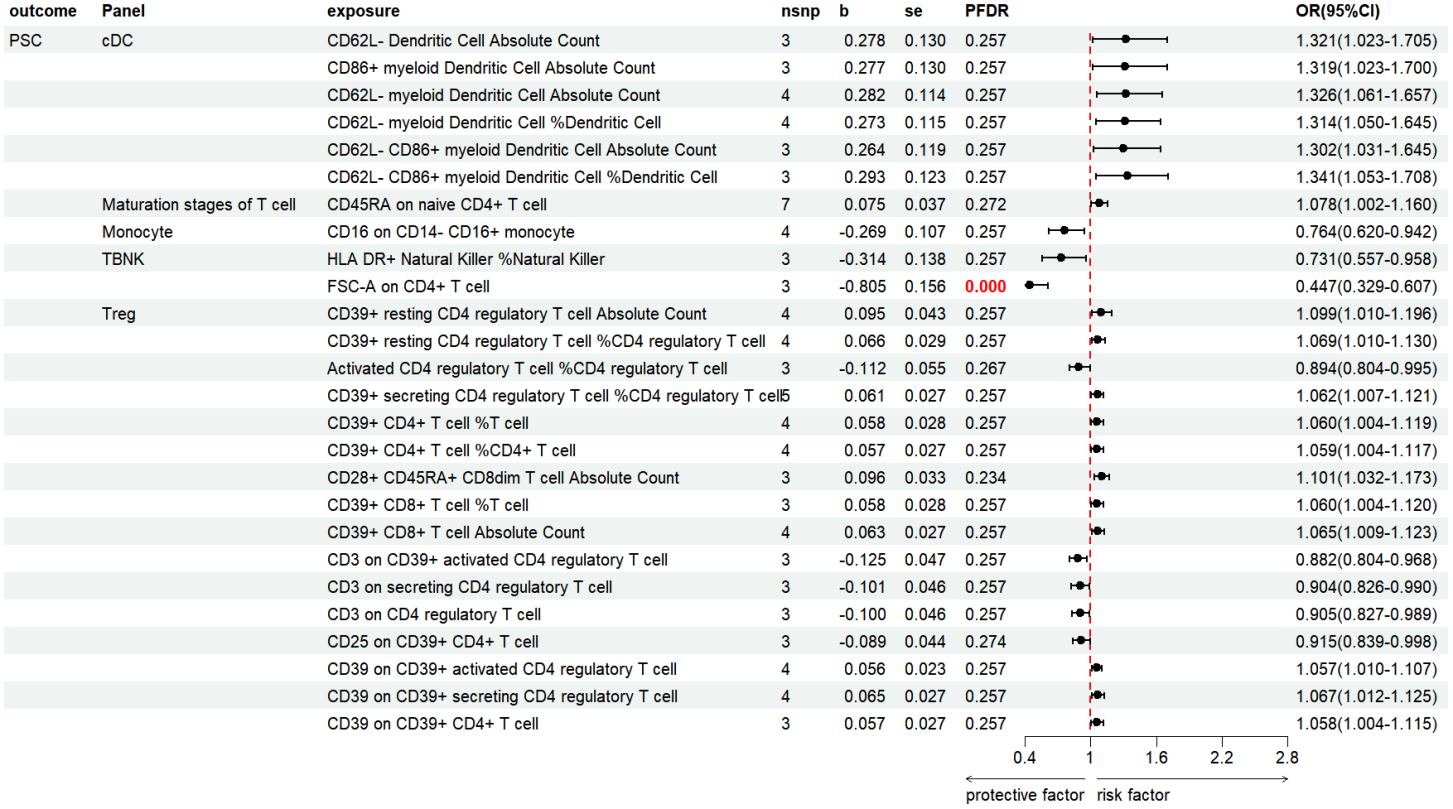
Forest plot shows the positive IVW-MR results of causal links between immune cell and PSC.

The results revealed that 26 immune cell phenotypes significantly influence PSC, as indicated by the IVW method. This includes 16Treg, 6cDC, 2TBNK, 1Monocyte, 1Maturation stages of T cell (Figure 7; Supplementary Table S7). By FDR correction (PFDR <0.05), we discovered one immunophenotype to be protective against PSC: *FSC-A on CD4+ T cell* (OR = 0.447, 95%CI 0.329 ~ 0.607, *p* = 2.530 × 10^−7^, PFDR = 7.104 × 10^−5^).

### 3.4 Reverse MR analysis

To investigate the causal relationship between the gut microbiome and AILDs further, we conducted a reverse MR analysis, treating AILDs as the exposure and the gut microbiome as the outcome. The results indicate a causal relationship between PBC and *Species.Odoribacter splanchnicus* (OR = 0.955, 95% CI 0.919–0.992, *p* = 0.020), and between PSC and *L histidine degradation I* (OR = 1.032, 95% CI 1.005–1.060, *p* = 0.020). These two gut microbiota will be excluded in subsequent mediation studies. However, no other significant causal relationships were observed, as detailed in Supplementary Tables S8–S10.

### 3.5 Mediation analysis result

To elucidate the potential mechanisms underlying the occurrence and development of AILDs, we carried out a mediation analysis to establish the causal pathways linking gut microbiota to AILDs through immune cells. This analysis builds on earlier findings that identified significant associations between certain gut microbiota and immune cells and AILDs, as detailed in our Supplementary Tables S11–S13. Initially, the causal relationships between specific gut microbiota and immune cells were assessed using two-sample MR. In the context of AIH, we identified 18 associations linking gut microbiota with immune cells. For PBC, we discovered 4 such associations, and for PSC, we detected 14 associations. MR mediation analysis revealed one immune cell type acting as a mediator in the pathway from gut microbiota to AIH. Notably, the *superpathway of L-tryptophan biosynthesis* to AIH appears to be mediated by the *CD14+ CD16+ monocyte Absolute Count*, with a mediation proportion of 17.47%. For PSC, one immunophenotype was identified as mediator. *Species. Ruminococcus obeum’*s influence on PSC may be mediated by the percentage of *CD62L-CD86+ myeloid Dendritic Cell*, accounting for 32.47% (Table 1).

**Table 1:** Results of two-step Mendelian randomization mediation analysis.

Exposure	Mediator	Outcome	X-M		M-Y		X-Y		Effect rate
			OR (95%CI)	*P*	OR (95%CI)	*P*	OR (95%CI)	*P*	
*Superpathway of L-tryptophan biosynthesis*	CD14+ CD16+ monocyte Absolute Count	AIH	0.856 (0.759,0.965)	0.011	0.700 (0.561,0.865)	0.001	1.380 (1.095,1.738)	0.006	17.47%
*Species.Ruminococcus_obeum*	CD62L-CD86+ myeloid Dendritic Cell %Dendritic Cell	PSC	0.730 (0.615,0.868)	0.0003	1.341 (1.052,1.708)	0.018	0.753 (0.593,0.956)	0.020	32.47%

X, exposure; M, mediator; Y, outcome; AIH, Autoimmune Hepatitis; PSC, Primary Sclerosing Cholangitis.

## 4 Discussion

In this study, we used genetic prediction to investigate how the abundance of gut microbiota and their pathways influence the development of AILDs. Our data were further refined to the species level, offering a more in-depth analysis compared to previous research and facilitating the exploration of potential mediating mechanisms involving immune cells. The large sample size and multivariable summary data of both gut microbiota and immune cells were utilized in MR analysis, yielding numerous positive associations with AILDs. Some of these findings are consistent with existing observational studies, thereby reinforcing their conclusions. However, other results contradicted previous findings, likely due to racial differences, warranting further investigation. Additionally, some novel findings provide new perspectives for future research.

Evidently, conclusions consistent with previous observational studies are of particular interest to us. *Roseburia* can upregulate tight junctions and enhance mucin production, thereby maintaining the integrity of the intestinal barrier and preventing the translocation of toxins such as Lipopolysaccharides (LPS) from the gut to the liver (Wei et al., 2020). Additionally, it increases anti-inflammatory cytokines, such as IL-22, effectively inhibiting the interaction between LPS and TLR4 and reducing hepatic inflammation pathways (Jiang et al., 2023), thus providing protection against AIH. By sequencing the highly variable V3-V4 region of the 16S rRNA gene in stool samples from patients with early-stage AIH and comparing these with samples from healthy individuals, we observed an AIH-associated enrichment of *Eubacterium* (Elsherbiny et al., 2020; Terziroli Beretta-Piccoli et al., 2022), which aligns with our study findings. The *species Eubacterium rectale* was also identified as a risk factor for PSC, yet it appears to be protective against PBC. In addition, we discovered that *Bacteroides* may reduce the risk of PBC. Evidence demonstrates that this bacterium can produce health-promoting phenylpropanoid derivatives and generate heparinase, facilitating the extraction of heparin and heparan sulfate from bacterial sources and categorizing it as a beneficial gut microorganism (Lv et al., 2016; Bohlmann et al., 2015). Similarly, fecal DNA sequencing in patients with PSC has revealed a significant reduction in the abundance of *Bacteroides* (Kummen et al., 2021). A reduction in *Ruminococcus* lowers Short-Chain Fatty Acids (SCFA) production, impairing intestinal barriers and potentially exacerbating PSC-related inflammation and immune responses through disrupted gut-liver interactions (Coufal et al., 2019). Prior research has identified the *Ruminococcaceae family* as protective against PBC. Our study advances this understanding, revealing that *Genus.Ruminococcaceae noname* and *Species.Ruminococcaceae bacterium D16* exhibit similar protective effects (Yang et al., 2024).

Gut bacterial pathway abundance refers to the presence and levels of specific metabolic and signaling pathways within the gut microbiome. Our research has discovered that the gut microbiota can play a protective role in AIH by participating in the key biosynthesis pathways of heme, polyamines, and riboflavin. Fibroblast growth factor 4 can increase the levels of heme oxygenase in ConA-induced AIH mice, thereby inhibiting ferroptosis in hepatocytes (Zheng et al., 2021). Tryptophan metabolites may serve as activators of the Aryl hydrocarbon Receptor (AhR), potentially inducing AIH-like pathology. Concurrently, the transcriptional activity of AhR is modulated by the Aryl Hydrocarbon Receptor Repressor (AHRR), which inhibits AhR signal transduction in Treg and Th17 cells, thereby facilitating the development of AIH (Xiang et al., 2024). Arginine residues at specific positions within the (Human Leukocyte Antigen)HLA-DRβ polypeptide are critical for determining genetic susceptibility to AIH. Additionally, studies demonstrated that the interaction between arginine and negatively charged amino acid residues on antigenic peptides facilitates salt bridge formation (Czaja and Donaldson, 2000). This enhances the stability and presentation of antigenic peptides to T-helper cells, thereby promoting immune activation and the continuation of the autoimmune response in AIH (Donaldson et al., 1994). The derivative of methionine, S-adenosyl-L-methionine (SAMe), exerts hepatoprotective effects through methylation and redox mechanisms. Notably, supplementation with SAMe significantly improves clinical symptoms in non-cirrhotic patients with PBC (Kilanczyk et al., 2020; Lai et al., 2022). Lipid A is a crucial component of the LPS discovered in the outer membrane of Gram-negative bacteria. It serves as the endotoxic component of LPS and plays a vital role in bacterial survival and virulence. Lipid IVA is a precursor in the biosynthesis of Lipid A and is critical for the proper formation of LPS (Paul et al., 2022). In a mouse model, knocking out L-histidine decarboxylase (HDC) led to reduced histamine levels, resulting in alleviated biliary damage and liver fibrosis. This suggests that histamine exacerbates PSC by intensifying inflammatory and fibrotic pathways (Kennedy et al., 2020).

Our study yielded findings that diverge from previous research. While earlier studies have suggested that *Ruminiclostridium 9* exerts a protective effect against AIH (Fu et al., 2023), and that the *Ruminococcaceae NK4A214* group increases the risk of AIH, our analysis did not confirm these associations. Notably, we identified *Ruminococcus obeum*, a member of the same family, as a potential risk factor for AIH. These discrepancies are likely attributable to differences in genetic backgrounds and interpopulation interactions across various regions. Geographic variations are a key determinant of gut microbiota diversity (Zhou et al., 2024). Immune homeostasis and genetic susceptibility are key mechanisms in the pathogenesis of AILDs (Tilg et al., 2022). In patients with PSC, there is a notable increase in naive-like CD4+ T cells in the liver compared to healthy liver tissue. Concurrently, another study indicates that reduced apoptosis in activated CD4+ T cells may play a role in the immunological dysregulation observed in PSC (Schoknecht et al., 2017; Poch et al., 2021). Our research suggests that FSC-A measurements on CD4+ T cells may act as a protective factor in PSC. The HLA genes are widely recognized as a genetic foundation for AILDs, albeit with some variations among different types. HLA-DR, a major histocompatibility complex class II molecule, participates in immune responses to extracellular pathogens by presenting antigens to helper T cells. Moreover, elevated expression of HLA-DR is usually linked to enhanced immune activation, which is essential for defending against pathogens. However, it can also lead to pathological autoimmune responses (Li et al., 2018; Higuchi et al., 2021). Our study indicates that HLA-DR expression on CD14-CD16+ monocytes and CD14+ CD16+ monocytes has a protective effect against PBC, while HLA-DR+ natural killer cells offer protection against AIH. Conversely, CD4 expression on HLA-DR+ CD4+ T cells increases the risk of PBC.

Recent advances in immunology and microbiology have uncovered complex interactions between the gut microbiota and the immune system, underscoring their significance in AILDs (Li et al., 2021; Liwinski et al., 2022). Yet, there are currently limited observational studies on how the gut microbiota influences AILDs through immune-mediated pathways (Li et al., 2022). To our knowledge, this is the first MR study to utilize immune cells as mediators to explore their role in the relationship between the gut microbiome and AILDs. Our findings are largely based on genetic predictions, demonstrating that the abundance of the *superpathway of L-tryptophan biosynthesis* in the gut microbiome correlates positively with the risk of AIH. This association is linked to the activation of the AhR by tryptophan metabolites (Xiang et al., 2024), with *CD14+ CD16+ monocyte Absolute Count* partially mediating this process. Genetically, the morphological parameters of immune cells play a central role in immunoregulation (Longhi et al., 2021). PSC often co-occurs with inflammatory bowel disease (IBD) due to interactions within the liver-gut-immune axis, which may result from the abnormal expression of gut-homing molecules in the PSC liver, thereby facilitating the transport of CD8 memory T cells between the gut and liver (Bozward et al., 2021). Consequently, fecal microbiota transplantation, widely used in IBD patients, may offer a novel therapeutic approach for PSC. Regulatory Tregs are crucial for maintaining the balance between the immune response to self-antigens and tissue damage caused by immune activation (Jeffery et al., 2016). Our study found that *Species.Ruminococcus obeum* may protect against PSC through mediation by *CD62L-CD86+ myeloid Dendritic Cell %Dendritic Cell*,providing new perspectives for future therapeutic targets for PSC (Li et al., 2019). Nevertheless, further research is required to validate these pathways.

However, this study has some limitations. First, the initial research lacked specific information such as age and gender, which are particularly relevant, provided that the incidence of AILDs appears to be associated with these characteristics according to epidemiological findings. The absence of this information hindered further subgroup analysis. Second, most of the GWAS data in this study were derived from individuals of European descent, limiting the generalizability of the findings to other racial groups. Lastly, although the MR method effectively assesses the causal relationship between exposure factors and outcomes, the credibility of these mediating mechanisms requires further validation through experimental and clinical studies.

## 5 Conclusion

This comprehensive analysis provides a clearer understanding of the complex interactions between the gut microbiome, immune cells, and the pathogenesis of AILDs, offering a valuable foundation for future research into targeted immune therapies for AILD patients. Nonetheless, additional basic and clinical research is essential to support these insights.

## Data Availability

The datasets presented in this study can be found in online repositories. The names of the repository/repositories and accession number(s) can be found in the article/Supplementary material.
